# Reasons underdetermination in meaningful human control

**DOI:** 10.1007/s10676-025-09858-x

**Published:** 2025-10-15

**Authors:** Atay Kozlovski

**Affiliations:** Delft University of Technology, Delft, Netherlands

**Keywords:** Meaningful Human Control (MHC), Reasons, Underdetermination, Incommensurability, Decision Support Systems, Moral Responsibility, Tracking and Tracing

## Abstract

The rapid proliferation of AI systems has raised many concerns about safety and responsibility in their design and use. The philosophical framework of Meaningful Human Control (MHC) was developed in response to these concerns, and tries to provide a standard for designing and evaluating such systems. While promising, the framework still requires further theoretical and practical refinement. This paper contributes to that effort by drawing on research in axiology and rational decision theory to identify a critical gap in the framework. Specifically, it argues that while ‘reasons’ play a central role in MHC, there has been little discussion of the possibility that, when weighed against each other, reasons may not always point to a single, rationally preferable course of action. I refer to these cases as instances of reasons underdetermination, and this paper discusses the need to address this issue within the MHC framework. The paper begins by providing an overview of the key concepts of the MHC framework and then examines the role of ‘reasons’ in the framework’s two main conditions - Tracking and Tracing. It then discusses the phenomenon of reasons underdetermination and shows how it poses a challenge for the achievement of both Tracking and Tracing.

## Introduction

The rapid proliferation of AI systems has raised many concerns about safety and responsibility in their design and use. The philosophical framework of Meaningful Human Control (MHC) was developed in response to these concerns, and tries to provide a standard for designing and evaluating such systems. While promising, the framework still requires further theoretical and practical refinement. This paper contributes to that effort by drawing on research in axiology and rational decision theory to identify a critical gap in the framework. Specifically, it argues that while ‘reasons’ play a central role in MHC, there has been little discussion of the possibility that, when weighed against each other, reasons may not always point to a single, rationally preferable course of action. I refer to these cases as instances of *reasons underdetermination*, and this paper will discuss the need to address this issue within the MHC framework.

This paper is structured as follows: Sect. 1 provides an overview of the key concepts of the MHC framework. Section 2 examines the role of ‘reasons’ in the framework’s two main conditions, Tracking and Tracing. Section 3 will discuss the phenomenon of reasons underdetermination (3.1) and show how it poses a challenge for the achievement of both Tracking (3.2) and Tracing (3.3).

### The framework of meaningful human control (MHC)

The concept of meaningful human control has emerged from the debate over the use of Lethal Autonomous Weapon Systems (LAWS) (Burri, 2018; Horowitz & Scharre, 2015). A variety of ethical and legal concerns have been raised against the deployment of these systems, such as difficulties in responsibility attribution (Matthias, 2004; Sparrow, 2007; Nyholm, 2023), failure to respect basic human dignity (Asaro, 2012), and non-compliance with international law (Eggert, 2024). The predominant response to these critiques has been to propose ways to ensure that a human actor remains, in some sense, ‘in’ or ‘on the loop’ (Cohen et al., 2023; Wagner, 2019). However, merely involving a human actor does not guarantee their effectiveness, particularly if they are expected to intervene or supervise over the actions of systems operating at a pace and scale far beyond human capabilities. Consequently, the literature has converged on the idea that ‘mere’ human control is insufficient unless it is ‘meaningful’ - hence the emphasis on meaningful human control.

While the concept of meaningful human control has remained relatively vague and contested in the legal domain (Davidovic, 2023; Ekelhof, 2019; Robbins, 2023), Santoni de Sio and van den Hoven ((Santoni, and Hoven, 2018)) have developed a theoretical philosophical framework that emphasizes the conditions that need to be met in order to achieve meaningful human control. Their framework draws inspiration from the literature on moral responsibility with particular emphasis on the theory of responsibility as guidance control as developed by Fischer and Ravizza (1998). In their book, Fischer and Ravizza outline two key conditions for attributing moral responsibility to an agent for a particular outcome: (i) Reasons-Responsiveness and (ii) Ownership.

The first condition, reasons-responsiveness, evaluates whether an agent is, in principle, capable of being both receptive and reactive to relevant reasons in a given situation. This assessment considers factors such as whether the agent was aware, or reasonably should have been aware, of pertinent reasons for acting, and whether the agent possessed the capacity to respond to these reasons when making a decision. If both receptivity and reactivity are satisfied, the agent meets the standard of reasons-responsiveness, qualifying them to be held morally responsible for the outcome. The second condition, ownership, examines whether the decision-making mechanism that led to the agent’s action can genuinely be attributed to the agent. This condition allows for the possibility of mitigating moral responsibility in certain circumstances, such as when an agent’s actions are influenced by external factors like drugs, manipulation by another party, or brainwashing.

In developing the MHC framework, Santoni de Sio and van den Hovan adapt the conditions of ‘moderate reasons responsiveness’ and ‘ownership’ so as to to apply to cases involving the use of AI systems. As a framework, MHC is designed to be ‘domain neutral’ such that, in principle, it can be applied to the evaluation of any AI system. To date the framework has been applied in a number of different design contexts (Mecacci et al, 2024) including Surgical Robotics (Ficuciello et al., 2019), medical diagnosis algorithms (Hille et al., 2023), Smart Home Systems (Umbrello, 2020), Autonomous Vehicles (Heikoop et al., 2019; Struik, 2021), Military Drone Systems (Steen et al., 2023), and more. As a framework, MHC looks at “what should, more generally, be the role of human control over AI systems, such that unreasonable risks are avoided, human responsibility does not evaporate, and there is a place to turn to in case of untoward outcomes” (Santoni de Sio 2024, 151). There are three key aspects which need to be clarified in order to see how the framework sets out to achieve these goals: (i) The framework’s holistic and socio-technical understanding of ‘what constitutes a system’, and its two key conditions of (ii) Tracking and (iii) Tracing which correspond to Fischer and Ravizza’s conditions of reasons responsiveness and ownership.

Starting with MHC’s conceptualization of a system, the framework uses this term to refer not merely to the AI algorithm or the hardware components of a technological apparatus, but also to the social and technical aspects in which the technology is embedded (van Diggelen et al., 2024). For example, when evaluating an Autonomous Vehicle (AV), the MHC framework considers not only the vehicle and its operators but also the broader traffic environment, including the associated social, legal, and political infrastructures (Mecacci & Santoni de Sio 2020, 106). Thus, in assessing how meaningful human control can be implemented within a ‘system’, the framework expands its analysis to encompass not just the technology itself but also various stakeholders and their direct or indirect influences on the system’s operation.

Turning now to the Tracking condition, Santoni de Sio and van den Hovan offer the following definition:

In order to be under meaningful human control, a decision-making system should demonstrably and verifiably be responsive to the human moral reasons relevant in the circumstances… That is, decision-making systems should track (relevant) human moral reasons. (Santoni de Sio and van den Hoven, 2018, p. 7)

According to this definition, for a system, in the sense explained above, to be under MHC, it must be capable of appropriately responding to, tracking, all relevant reasons in a given situation. For example, in the case of an AV, to meet the Tracking condition of MHC, the AV system must be able to: (i) identify all relevant reasons in a given situation; (ii) weigh those reasons against each other; and (iii) determine which action to take based on those reasons. For instance, while my plan as a passenger might be to arrive home in time to watch the football game, I obviously intend to do so safely and would not want the AV to take sharp turns at high speed. Similarly, even if I don’t mind driving slightly over the speed limit or ignoring a ‘Stop’ sign that is often overlooked, the AV should not only track my driving habits and preferences but also adhere to traffic laws and road etiquette. Therefore, a primary challenge in achieving the Tracking condition is determining whose and which reasons the system should track and how to resolve cases where those reasons conflict (van den Hoven 2012), van de Poel, 2015).

Shifting to the Tracing condition, this condition emphasizes the need to ensure that some human agent meets the required conditions to be held morally responsible for any potential harm caused by the AI system. The Tracing condition is achieved within a system if at least one human agent “has the capacity to (i) understand the capabilities of the system while at the same time (ii) appreciating their own moral responsibility for its behaviour” (Mecacci and Santoni de Sio, 2020. P. 105). While (ii) seems relatively straightforward, there is significant room for interpretation regarding the meaning of (i). What does it mean to ‘understand’ the capabilities of the system? Is it sufficient to know how the system typically behaves, or does one need expert knowledge of the system’s inner logic? MHC intentionally remains vague on this issue to allow for adaptation of the conditions to different design contexts.

Take, for example, the widely discussed 2018 fatal car crash involving an Uber-operated AV and the pedestrian Elaine Herzberg. The aftermath of this incident raised many questions about who should be held responsible for the tragic accident - was it the designers of the AV, the software engineers, the human test driver present at the time, the legislators who approved the use of the AV on public roads, Uber itself, or all of the above (Kiener, 2024)? Such situations are often discussed as potential ‘responsibility gaps’ (Matthias, 2004, Santoni de Sio & Mecacci 2021, Nyholm, 2023) - cases in which several parties might be considered responsible, yet none seem to meet the standard conditions of knowledge and control needed to be held morally responsible for the AV’s actions.

Although the MHC framework remains neutral on the theoretical possibility of ‘responsibility gaps’, a topic contested in the literature (Tigard, 2020; Hindriks & Veluwenkamp, 2023), it still aims to ensure that a system is designed to establish clear lines of responsibility and that this responsibility can be properly and effectively acted upon when necessary. In the case of the AV crash, for instance, Tracing would not be achieved by arbitrarily designating the safety driver or the software engineer as responsible for the AV’s actions. Instead, Tracing requires that the system be designed so that the responsible agent has the relevant training, both physical and mental, to undertake this role and that the system is structured to make it technologically feasible to act on this responsibility (Santoni de Sio and van den Hoven, 2018).

## ‘Reasons’ in MHC

The previous section provided a brief overview of the Tracking and Tracing conditions in the MHC framework. In this section, we aim to show that reasons play a central role in both operationalizing and fulfilling these two conditions. For the Tracking condition, this link is straightforward, as it is directly defined in terms of a system’s reasons-responsiveness. In contrast, the connection between reasons and Tracing is more complex, emerging only once the role of reasons in responsibility attribution is clarified. This discussion lays the groundwork for Sect. 3, where we examine the phenomenon of reasons underdetermination and explore its implications for both Tracking and Tracing.

### Reasons in tracking

Tracking, according to Santoni de Sio et al. (2023, 593) “focuses on the nature of the relationship between human controllers and controlled intelligent systems”. This relationship is framed in terms of the system’s ‘reasons-responsiveness’, with the goal of tracking being to assess or ensure that a system’s output responds appropriately to “human moral reasons relevant in the circumstances” (Santoni de Sio & van den Hoven, 2018, 7). This raises key questions: How does MHC define a moral reason? Which reasons are relevant in a given situation? And what does it mean to respond appropriately? While I will not answer these questions in detail, a brief overview will set the stage for our next section on how reasons underdetermination can pose a challenge to achieving tracking.

To begin, it is worth noting that the literature on MHC often uses the concept of a ‘moral reason’ as a placeholder for a variety of related terms, including goals, plans, norms, intentions, and values (Mecacci & Santoni de Sio 2020). While this broad range of concepts might initially seem problematic, it can actually be understood as a way for MHC to remain flexible across different design contexts. In this sense, ‘reasons’ functions as a unifying term that encompasses all these concepts without excluding factors that may be important in specific situations. For example, an autonomous vehicle might be programmed to follow my instructions regarding a destination while ignoring a goal of causing a traffic jam by driving slowly in the left lane. In some design scenarios, values and norms may take priority, whereas in others, preferences and goals may be more relevant. MHC addresses this by treating all of these factors as ‘reasons’ and requiring the system to assess their relevance and importance in each case.

This brings us to the second question: which reasons does MHC consider relevant for tracking? This can be understood in two ways. First, there is the question of what ‘types’ of reasons a system should track, and second, which specific reasons are relevant in a particular situation. Regarding the former, this might involve distinctions such as normative versus motivational reasons or agent-relative versus agent-neutral reasons (Veluwenkamp, 2022), raising the question of whether tracking applies to one or all of these categories. As for the latter, specific reasons, it is challenging to define relevance outside a particular design context. Nevertheless, any system must have a method for narrowing down the reasons it considers relevant. For example, while the fact that it is raining may be a relevant reason for an autonomous vehicle to adjust its speed, it would not be relevant when providing a career recommendation.

Lastly, what does it mean for a system to be ‘appropriately responsive’ to reasons? This concept has both technical and normative dimensions. Technically, it means the system makes decisions based on identified relevant reasons rather than random or arbitrary processes. Normatively, it involves assessing the significance of each reason and making decisions through an all-things-considered evaluation. For example, a hiring recommendation shouldn’t rely solely on Abigail’s experience; while relevant, the decision should weigh all factors to determine her overall fit for the role. Appropriate responsiveness, therefore, entails considering the balance of reasons, not just isolated ones.

Thus, to summarise, achieving Tracking requires that a system be able to identify the relevant reasons for action, assess their significance, and act on the basis of their overall merits. In this sense, Tracking can be understood as a form of the ‘alignment problem’ (Bostrom, 2016; Russell, 2019; Christian, 2020; Gabriel, 2020), solved when the system is capable of identifying, evaluating, and responding appropriately to relevant reasons. While Tracking is also closely connected to the attribution of moral responsibility to humans for a system’s actions, and thereby to the mitigation or elimination of responsibility gaps, I will not elaborate on this point here, but will address the issue of responsibility gaps in the discussion of Tracing in the next section.

### Reasons in tracing

As discussed in Sect. 1, MHC is highly concerned with the possibility that the use of (semi)autonomous AI systems will create responsibility gaps. Before discussing this in any detail we first need a basic grasp of the multifaceted notion of responsibility. Although we will only scratch the surface here, it is important to highlight three differing notions or aspects of moral responsibility (Talbert, 2022): Accountability, Answerability, and Attributability. To help clarify these three types of responsibility we will apply each to the case of Herbert the HR executive:

Since his promotion to HR executive, Herbert has held final authority over the company’s hiring decisions. He conducts the interviews, evaluates and ranks the candidates, and signs off on the final choice. For one open position, Herbert reviewed a large pool of applicants and ultimately offered the role to Abigail, while declining April. A few days later, April filed a formal complaint alleging that her rejection was due to her ethnicity. In response, an investigatory panel has been convened to examine these claims. After a brief investigation the panel concluded that Herbert was responsible for this discrimination.

So what did the panel actually conclude? The answer depends on which notion of responsibility they were considering. Beginning with responsibility as **Accountability**, this concept concerns whether it is appropriate for an agent to be the subject of reactive attitudes such as blame or praise. As Shoemaker explains, “To be accountability-responsible is just to be susceptible for being the appropriate target of sanctions (or perhaps rewards, in positive cases)” (Shoemaker, 2011, p. 628). In other words, claiming that Herbert is accountability-responsible means the panel has concluded that he met certain conditions of knowledge and control, he is not exculpable, and is therefore blameworthy, warranting treatment in accordance with that judgment.

Second, if the panel concluded that Herbert was responsible in terms of **Answerability**, they would be claiming that he is the agent in a position to provide an explanation or justification for the action taken (Zeiser, 2024, p. 4). That is, the panel would note that Herbert conducted the interviews, ranked the applicants, and had sole discretion over the final hiring decision. In this case, they might require Herbert to explain his decision and, based on that explanation, also determine whether he is accountable for the discriminatory outcome.

Finally, the panel might conclude that Herbert was responsible in terms of **Attributability**. This notion of responsibility is not concerned with identifying the correct agent to punish, nor with clarifying the reasons for an action, but with the agent whose action it is and the extent to which the action reflects their practical identity and value commitments. As Watson explains:

This brings out the way in which aretaic appraisal involves an attribution of responsibility. To adopt an end, to commit oneself to a conception of value in this way, is a way of taking responsibility. To stand for something is to take a stand, to be ready to stand up for, to defend, to affirm, to answer for. Hence one notion of responsibility - responsibility as attributability - belongs to the very notion of practical identity. (Watson, 1996, p. 234)

Thus, concluding that Herbert is responsible in this sense means passing judgment on Herbert’s identity as a discriminating individual.

With these notions of responsibility in hand, we now turn to the Tracing condition. Recall that Tracing involves two key requirements: (i) a human agent must understand the system’s capabilities, and (ii) they must recognize their moral responsibility for its actions. Achieving these requirements within a socio-technical system depends on numerous contingent factors and the specific context. For instance, implementing Tracing in a system for screening job applicants will differ significantly from achieving it in other contexts, such as an autonomous vehicle or an LLM-powered note-taking tool in a doctor’s office. Consequently, the operationalization of Tracing may focus on many different aspects of the socio-technical system, including user training procedures, feedback and contestation mechanisms, and forms of human oversight or involvement. However, since our aim in this section is to highlight the role of reasons in Tracing, the analysis that follows will concentrate on challenges related to responsibility attribution in the context of AI-based decision support systems (DSS).

What is a DSS? Unlike AI systems designed to replace human agents, DSSs are intended to enhance and support human decision-making. First developed in the 1960 s (Arnott & Pervan, 2005), these systems have since benefited from advances in technology, including modern machine learning methods, increased computing power, and access to vast datasets. DSSs are promoted as tools that can improve the quality and speed of decisions by overcoming human limitations such as biases, inconsistencies, limited time, and errors (Kahneman et al., 2021; Sunstein, 2024), as well as by detecting patterns and correlations that might escape human notice. Such capabilities have led to their adoption across a wide range of domains, including the U.S. judicial system’s use of COMPAS to assess recidivism risk (Angwin et al., 2016), the Dutch system for detecting fraudulent child-benefits applications (Amnesty International, 2021), the Viogen system used by Spanish police to evaluate the risk of repeated gender-based violence (Castro-Toledo et al. 2023), and military systems such as Lavender and Maven, which provide targeting recommendations (Kozlovski, 2024).

Although DSSs have grown increasingly popular, critics caution that they are far from perfect, raising concerns about algorithmic bias, human deskilling, automation bias, and potential responsibility gaps (Vallor, 2015; Dastin, 2018; French & Lindsay, 2022; Wachter, 2022). It is this last issue, the challenge of attributing responsibility, which will be our focus. In particular, many authors warn about the risks posed by black-box DSSs (Rudin, 2019) which, due to their complex architectures, sophisticated algorithms, and reliance on massive datasets, often make it impossible to undestand how or why a system arrived at a specific recommendation.

To illustrate the problem for responsibility attribution, consider Baum et al.’s (2022) version of the Herbert scenario discussed above. In this case, Herbert’s company introduces a DSS to assist him throughout the hiring process:

Imagine that Herbert decides to exclude April’s application because the hiring system recommended doing so. Imagine further that the system’s recommendation is due to its bias against Black female applicants, but that, since it is an accredited system, Herbert justifiably believes that it has no such problems. (Baum et al. 14)

Now, assuming the DSS is indeed a black-box system that does not allow Herbert access to the reasoning behind its recommendations, can Herbert be held morally responsible for the discrimination against April? Once again, the answer will depend on which notion of responsibility is being applied.

Starting with the question of accountability, Baum et al. (2022) argue that, as described, Herbert could not be held accountable for the discrimination against April because his reliance on the DSS means he no longer satisfies the epistemic (knowledge) condition for responsibility:

“An agent is morally responsible for her action or decision only if she has sufficient epistemic access to it. That she has sufficient epistemic access to it entails at least that she is in a position to know the action under relevant descriptions” (Baum et al., 2022, p. 13).

Accordingly, although Herbert knows that he is rejecting April’s application, the DSS provides no explanation for its recommendation. Consequently, he does not have enough epistemic access to recognize that his action results in discrimination, and is therefore not directly morally responsible, understood as **accountability**, for the outcome.

Second, can Herbert be considered responsible for the discrimination in terms of **answerability**? Again, the answer appears to be no. While Herbert may be able to explain why he followed the DSS’s recommendation to reject April’s application, he cannot provide any insight into why the system produced a discriminatory outcome. By relying on the DSS without understanding its reasoning, Herbert cannot offer adequate reasons for the rejection and effectively becomes a messenger delivering the system’s verdict. The same reasoning applies to **attributability**. It would be highly unfair to conclude that Herbert is racist or discriminatory based on this case. He had no access to the DSS’s reasoning, and the decision to exclude April was not grounded in any value commitment on his part that could be attributed to his practical identity.

The story of Herbert and the DSS highlights how responsibility gaps can emerge within a socio-technical system. Because Herbert lacked an understanding of why the system produced its recommendations, it was difficult to hold him directly morally responsible, in any of the three senses discussed, for the discrimination against April.

Abstracting from this specific thought experiment, theorists on responsibility gaps emphasize that just as there are multiple forms of responsibility, there are likewise multiple types of corresponding responsibility gaps. For example, Zeiser (2024), building on Watson’s theory of responsibility as attributability, argues that attributability gaps can arise, as demonstrated in the Herbert case. Similarly, Santoni de Sio (2024, 121) identifies four types of responsibility gaps that the MHC framework seeks to address: the culpability gap, the moral accountability gap, the public accountability gap, and the active responsibility gap. The first three correspond closely to the notions of responsibility as accountability and answerability.

To bring this section to a close, how can Tracing attempt to address these potential responsibility gaps and what is the role of reasons in enabling this? While there are many approaches to mitigating and addressing responsibility gaps, appealing to explainability so as to enable a human user to understand and evaluate the reasons behind a system’s actions, especially in the context of DSSs, seems to be the most straightforward and a common approach. For instance, Floridi et al., in developing their ‘ethical framework for AI’, discuss the importance of system explicability (their term for a combination of transparency, accountability, understandable and interpretable systems) and recommend that we must “Develop a framework to enhance the explicability of AI systems that make socially significant decisions. Central to this framework is the ability for individuals to obtain a factual, direct, and clear explanation of the decision-making process, especially in the event of unwanted consequences” (Floridi et al., 2018, p. 702).

Building on this position, others such as Baum et al. (2022) and Zeiser (2024) also discuss the importance of explainability for moral responsibility. Baum et al., for instance, argue that “Just like human experts would provide reasons for their recommendations, so should decision support systems” (Baum et al., 2022, 17–18). In contrast, Santoni de Sio offers a slightly more nuanced perspective, acknowledging the value of explainability but cautioning against overreliance on it, which risks a form of techno-solutionism:

Theorists identified transparency and explainability of algorithms and AI as important elements to safeguard the ‘traceability’ of human responsible agents and, consequently, a fair attribution of moral responsibility. I believe that algorithmic explainability, though constituting one interesting element in a complex strategy to address the responsibility gaps, is neither a sufficient nor a necessary condition to address them. (Santoni de Sio, 2024 136).

And so, to conclude, this section has shown that reasons play an important role in Tracing in that they can be crucial for moral responsibility attribution, understood in the three senses of accountability, answerability, and attributability. When AI systems are involved, especially DSSs, avoiding responsibility gaps often depends on the human agent’s access to the system’s reasons for action and their ability to understand why a recommendation was made. Although Tracing should not be reduced merely to an explainability problem, I have tried to show in this section that in many cases this will be a common and effective method to attempt to operationalise and achieve Tracing.^1^

## ‘Reasons underdetermination’

In the previous two sections, we explored the MHC framework and the crucial role that reasons play in the conditions of Tracking and Tracing. In this section, I will focus on the phenomenon of reasons underdetermination and the challenge it poses to fulfilling the conditions of MHC. I will begin by discussing the existing literature on reasons underdetermination and rational decision-making, followed by an analysis of how this issue impacts the Tracking and Tracing conditions.

### When reasons underdetermine choice

Reasons underdetermination is a philosophical term of art which I will use to refer to cases in which the reasons supporting different options (A, B) in a decision making situation fail to establish that one is better than the others or that the two are equally good options for choice. Before discussing the relevance of this phenomenon to MHC, it will be helpful to first clarify the relationship between reasons and rational decision-making in general. Although the following discussion will be relatively brief, considering the vast amount of literature on these issues, it should provide enough background information for clarifying the phenomenon of reasons underdetermination.

To start, in this paper I will be working within a comparativist framework for rational choice which holds the following claim:

“comparative facts about the strengths of the reasons for and against the options… is that in virtue of which a choice is rational in that situation”. (Chang, 2016a, p. 215)

In other words, the rationality of a decision depends on the strength of the reasons supporting it, compared to the reasons opposing it or favoring an alternative. Additionally, as we briefly discussed in the previous section, rational choice is determined by an all-things-considered evaluation of the reasons in a comparison, where ‘all-things-considered’ is used as a placeholder for a predefined set of criteria called the ‘covering consideration’ (V) of the comparison (Chang, 2001). That is, A cannot be ‘better than’ B *simpliciter*; it is always relativised to a specific V. Although we sometimes say, for instance, that Sushi is better than Pizza, for this statement to have any meaning it must be understood as shorthand for ‘Sushi is better than Pizza’ in terms of some V - e.g. ‘tastiness’.

Comparativism holds that there is a neat isomorphism between all-things-considered judgments and rational choice: If A is better than B (all-things-considered), then one rationally ought to choose (A) Likewise, if A is worse than B (all-things-considered), then one rationally ought to choose (B) And if A and B are equally good (all-things-considered), then one rationally ought to pick either A or B by using a random selection process (Ullmann-Margalit & Morgenbesser, 1977). Thus, reasons form the basis for rational decision-making by indicating which option an agent should select according to the balance of the weight of the relevant reasons.

What then are cases of reasons underdetermination? The Philosopher Joseph Raz describes this best in what he calls ‘the basic belief’:

that most of the time people have a variety of options such that it would accord with reason for them to choose any one of them and it would not be against reason to avoid any of them (Raz, 1999, p. 100).

Raz defines the concept of ‘Reason’ as the “general capacity to recognize and respond to reasons” (Raz, 2011, P. 92). As such, the ‘basic belief’ holds that reasons make options eligible for choice but often fail to indicate that one course of action or choice is the best or rational thing to do. So how does this ‘basic belief’ fit with comparativism? Does this mean that Raz believes that most of our available choices are ‘equally good’ and we simply randomly select from among the set of acceptable options? No.

Like De Sousa (1974) and Derek Parfit (1986) before him, Raz argues that the interplay between reasons for action does not always result in or should be limited to the three basic evaluative relations of ‘better than’, ‘worse than’, or ‘equal to’. Instead, Raz argues that competing options will often be incommensurate:

A and B are incommensurate if it is neither true that one is better than the other nor true that they are of equal value. (Raz, 1988 322)^2^

It is essential to differentiate Raz’s use of ‘incommensurate’ from the concept of ‘incommensurability’. Incommensurate options, in Raz’s terms, are an instance of what we are calling reasons underdetermination. In contrast, incommensurability refers to a lack of a common unit of measurement for comparing two options. For example, we can compare the volumes of two cylinders using the formula πr²h, making them commensurable. However, when assessing two immigration policies based on ‘Justice’ they are incommensurable because there is no unit, like a ‘Justone’, to quantify Justice. Additionally, incommensurability should not be confused with the complete inability to compare the option (Chang, 2015). Even without a unit of measurement we can argue that Policy A, which suggests rejecting immigration requests based on the name of an applicant, is less just than Policy B, which advocates for fair and unbiased quotas.

Returning now to Raz and incommensurate options, he offers the following example to describe such cases:

Let us take as our example the case of a person who has to choose between two options. The one will irrevocably commit him to a career in law, the other will irrevocably commit him to a career as a clarinetist… assume for example, that neither is better than the other. It hardly needs arguing that in that case they are incommensurable. The suggestions that they are of exactly the same value cannot be entertained seriously. (Raz, 1988, 332.)

Many other examples have been discussed in the literature, ranging from low-stakes choices like choosing between coffee and tea (Chang, 2002, p. 669) to high-stakes decisions such as choosing a life-saving operation that prolongs life but causes severe pain versus forgoing it for a shorter, pain-free life (Sinnott-Armstrong, 1985, p. 327). The takeaway here is that reasons underdetermination is a common and widespread occurrence in evaluative judgments and normative decisions making.

In the literature, we find a variety of explanations for why reasons can sometimes underdetermine choices. Raz, for instance, attributes this to an ‘incomplete’ definition of how criteria contribute to a value, stressing normative incompleteness over a mere lack of relevant knowledge (Raz, 1988, p. 326). Others have highlighted different notions of vagueness (Broome, 1997) - whether epistemic (Flanigan and Halstead, 2018), deontic (Barnes, 2010), or semantic (Wasserman, 2004) - which suggest that choices underdetermined by reasons may involve borderline cases, where it’s neither definitively true nor false that A is better than B. Another perspective focuses on the qualitative nature of such choices. Chang, for example, illustrates that “a boring life with financial security is qualitatively very different in well-being from an exciting one that involves a hand-to-mouth existence” (Chang, 2016b, p. 405). This focus on qualitative comparisons ties back to the idea of incommensurability, where precise quantitative evaluations are often impossible due to the lack of a unit of measurement.

So how do we choose between options that are underdetermined by reasons? While this is a widely debated issue, I will focus on two prominent views: Precisification (Fine, 1975) and Commitments (Chang, 2017). Those who believe vagueness is the root cause of reasons underdetermination often support a precisification method, where a more precise version of the comparison is arbitrarily selected. This perspective holds that vagueness arises from multiple legitimate interpretations of the covering consideration; and by choosing one of these interpretations, you can resolve the ambiguity. For example, if it’s vague whether Alex is ‘balder than’ Bobby, this might result from there being two eligible definitions of the predicate ‘bald’ or the comparative ‘balder than’: Bald1 and Bald2. Accordingly, reasons underdetermination occurs when it’s unclear which definition applies, and selecting one will clarify the situation.

Alternatively, Chang has rejected such vagueness views in favor of her own theory of Parity which claims that there exists a fourth value relation, being on-a-par with, which complements the traditional three relations of ‘better than’, ‘worse than’, and ‘equal to’ (Chang, 2002). When confronted with on-a-par alternatives Chang has argued extensively that a rational agent can make a personal rational commitment towards one of the options and in so doing create new reasons to favor that alternative and make it the option which is most rational to choose.

Parity is special because it is the relation that allows us to make it true, through an exercise of our normative powers, that we have most all-things-considered reasons to do one thing rather than another. It is the point at which we come into our own as self-governing agents. (Chang, 2017, p. 19)

For Chang, instances of reasons underdetermination, when options are on-a-par, represent identity forming opportunities; reasons can only take us so far by indicating which are acceptable courses of action, it is commitments that are made in those moments which enable autonomous rational agents to shape their own path through life.

Although these sketches provide only a rough outline of two possible approaches to decision-making under conditions of reasons underdetermination, they are sufficient for our purposes here, since the analysis in the following sections is concerned with showing how reasons underdetermination challenges the MHC framework. The task of developing possible strategies to resolve or accommodate this problem, however, must be left to future research.

### Reasons underdetermination in tracking

In Sect. 2.1, we explained that the Tracking condition can be understood as a type of alignment problem, where the challenge is to determine whose reasons are relevant, which of those reasons matter, and how much weight each should carry in guiding a system’s decisions. Put simply, for a system to achieve Tracking it must do two things: identify the relevant reasons and determine a course of action or output on the basis of their relative weight. In this section, I examine three approaches discussed in the MHC literature that attempt to address these tasks, and I show that none adequately account for the problem of reasons underdetermination.

The first approach I want to discuss appears in Veluwenkamp (2022) and argues that tracking should focus exclusively on normative, rather than motivating, reasons. This approach answers the question, ‘Which reasons should a system track?’, by distinguishing between different types of reasons. This distinction, between normative and motivating reasons, is central in the literature on practical reasoning and is perhaps best captured by Jonathan Dancy, who explains that there are “good reasons and those for which the agent acted” (Dancy, 2000, p. 1). Accordingly, each type of reason addresses a different question:

- **Normative Reasons**: Did you have ‘good’ reason(s) to act as you did?
- **Motivating Reasons**: Which reason did you act for?

A key difference is that motivating reasons are categorical - an agent either acted for a reason or did not - whereas normative reasons are graded - they can vary in strength and are typically weighed against each other.

Veluwenkamp argues that Tracking must “spell out reason-responsiveness in terms of normative reasons” (Veluwenkamp, 2022, p. 50). At first glance, this seems intuitive, after all, why would we want a system to consider *bad* reasons when making decisions or recommendations? Yet, while tracking normative reasons may be appropriate in some contexts, in others it risks being improper or even unacceptable. Consider an AV: if it tracks only normative reasons and disregards motivational ones, it might refuse to cooperate with a plan for revenge, such as running over a neighbor’s flower bed. This seems entirely sensible. But there are also situations where ignoring non-normative, motivational reasons, appears wrong. Take the case of a gambling addict instructing their AV to drive them to a casino to gamble away their child’s college fund. Normatively, this is a terrible decision. But should the AV really refuse the request? The challenge, then, is how to distinguish between cases where rejecting non-normative reasons seems justified and those where it seems inappropriate.^3^

This brings us to a second approach in the literature, which proposes the creation of an “explicit moral operational design domain (MODD)” (Siebert et al., 2022). In the case of AVs, an ODD already defines “a set of contextual conditions under which a driving automation system is designed to function” (ibid., p. 245). An MODD would extend this idea by setting explicit moral boundaries, thereby narrowing the range of reasons the system must consider. For example, an MODD could prohibit the AV from driving onto lawns or off designated roads, except in emergencies. It could also specify that the system should not evaluate reasons tied to a passenger’s choice of destination. This way, the AV would not refuse to drive the gambler to the casino, even if the decision is normatively objectionable. By establishing an MODD, we could complement Veluwenkamp’s proposal by restricting the normative reasons tracked to a predefined set of relevant cases.

The third approach to the tracking problem, developed by Mecacci and Santoni de Sio, introduces what they term a *‘Proximity Scale of Reasons’* (Mecacci & Santoni de Sio 2022). Building on theories from the philosophy of action and practical philosophy, their proposal adapts the distinction between ‘proximal’ and ‘distal’ intentions to construct a two-dimensional framework for how reasons should guide a system’s behavior. Within this framework, each reason is assigned a ‘proximity value’, which in turn allows the system to apply algorithmic rules for prioritizing among competing reasons.

For example, a simple algorithmic principle they propose for an AV might be:

1. The system should respond to a proximal reason *if and only if* it does not conflict with a more distal reason.
2. The system should respond to the most proximal reason that satisfies (1). (Mecacci and Santoni de Sio, 2022, 112.)

Mecacci and Santoni de Sio illustrate this with a thought experiment. Lucy, driving home, wishes to take manual control of her AV. Because her desire to drive manually has a high proximity value, the AV initially grants her control. A few minutes later, however, the AV’s sensors detect that Lucy is failing to slow down despite an obstruction in the road. Although the system has so far acted on Lucy’s proximal reason, it is also tracking a more distal reason tied to safety. Since the algorithm stipulates that no proximal reason can override a more distal one, the AV intervenes and takes back control to prevent a collision. In this way, the proximity scale, together with an algorithmic decision rule, enables the AV to determine which reason to prioritize in a given situation.

Although each of the three approaches discussed in this section offers valuable insights into how Tracking might be operationalized, none appears to address the challenge posed by reasons underdetermination. To begin, consider Veluwenkamp’s proposal to focus exclusively on normative reasons. While appealing in its clarity, this approach overlooks the fact that normative reasons are often precisely those that underdetermine choice. For example, consider the case of choosing between a career in law or one as a clarinetist. In some circumstances, normative reasons may clearly favor one option, but in many cases the relevant normative reasons underdetermine which career ought to be pursued. Thus, even if a system is designed to track only normative reasons, it still requires a method for addressing cases in which those normative reasons underdetermine the choice at hand.

Turning to the second approach, the MODD improves upon Veluwenkamp’s proposal by excluding certain reasons from the system’s consideration. However, it still fails to recognize that even within the MODD there can be cases in which reasons do not rationally determine the choice at hand, meaning the problem of reasons underdetermination persists.

Finally, Mecacci and Santoni de Sio’s *Proximity Scale of Reasons* offers a further refinement over the previous two approaches. Not only could it potentially integrate elements of both, by, for instance, limiting the system to track only normative reasons within the MODD, but by assigning a ‘Proximity Value’ to each reason, the system could in principle resolve instances of reasons underdetermination. How so? If the ‘Proximity Value’ is understood as a cardinal score assigned to each reason, this would imply full commensurability among reasons, allowing the system to determine whether one reason’s proximity, weight, or importance is greater, lesser, or equal to any other.

Lastly, Mecacci and Santoni de Sio’s ‘Proximity Scale of Reasons’ offers an improvement on the two previous approaches. Not only could it potentially incorporate both approaches by, for instance, designing the system to track only normative reasons that are in the MODD, but by producing a ‘Proximity Value’ for each reason within the MODD the system could ostensibly avoid instances of reasons underdetermination. How so? If by ‘Proximity Value’ Mecacci and Santoni de Sio imagine some kind of cardinal value score assigned to each reason, then this would entail full commensurability between those reasons and so we could easily determine whether the proximity/weight/importance of one reason is greater, lesser, or equal to any other.

However, Mecacci and Santoni de Sio make an intriguing remark at the end of their discussion of the Proximity Scale of Reasons:

Our general claim is that vehicles that are under MHC should also respond to some distal reasons of their owners/drivers as well as to some (distal) reasons of other agents in society, as reflected in some moral and legal norms. Which of these reasons specific systems should track remains a normative question on which reasonable persons and policy-makers may disagree. (Meccaci and Santoni de Sio 2020, 113)

I find this statement noteworthy because, although it was clearly not written with reasons underdetermination in mind, it comes remarkably close to touching on the very challenge we have been discussing. The idea that reasonable people can disagree over whether a system should track reason A or reason B mirrors what Raz highlighted in his concept of the ‘basic belief’. To be clear, Mecacci and Santoni de Sio likely frame the point this way to avoid making substantive commitments, for example, that AVs should prioritize safety over flexibility. Nevertheless, one could interpret their position as implying that, should such a substantive commitment be made, it would then be possible to create a proximity scale and assign a proximity value to all relevant reasons the system ought to track.

This interpretation, if correct, seems to expose the fact that the proximity scale of reasons was not designed with reasons underdetermination in mind. Two brief quotes illustrate that the approach relies on assigning value to different reasons. First, in their toy algorithmic principle, the second clause states that “The system should respond to the most proximal reason that satisfies (1)” (Mecacci & Santoni de Sio, 2022, 112). To determine which reason is more proximal, the system must measure reasons against one another. Second, they write that “To model this complex relation, and substantiate the tracking criteria, we propose a model… where human reasons are ordered in a scale with respect to how closely they influence a system’s behaviour” (Mecacci & Santoni de Sio, 2022, 109). However, the very suggestion that we can and should assign a Proximity Value to relevant reasons is in conflict with the idea that many comparisons involve incommensurability. Recall that incommensurability is the claim that a comparison lacks a unit of measurement by which to precisely evaluate alternatives options. As such, in order to assign a ‘Proximity Value’ to different reasons we would need to manufacture a unit of measurement which may, normatively speaking, not exist.

This problem can be illustrated with a hypothetical example involving soldiers mutinying, a scenario that has occurred repeatedly throughout history. Imagine a group of soldiers rebelling due to grievances such as unpaid wages, poor conditions, or extended service. Historically, such uprisings have been addressed in many different ways: some resolved through negotiation, others suppressed with harsh punishments, including executions in extreme cases. Deciding how to respond involves numerous factors: the soldiers’ actions, the current condition of the unit, the timing, and the officer in command, among others. Even with these considerations, significant discretion remains with the commanding officer in determining the appropriate response and any punishment. Cases like these are rarely reducible to rigid rules; they require weighing multiple factors and navigating difficult trade-offs between competing values, such as justice and expedience, or deterrence and reconciliation.

With this example in mind, we must ask: could a DSS be designed to assist the officer in charge in making the “best” decision in such cases? Of course technically we could do this - we could invent a method for assigning a precise weight, call it a proximity value, to each relevant aspect of the case, call them the reasons, and then calculate our way toward an ideal solution. Hopefully, the absurdity of such an approach is evident. Such cases seem to be paradigmatic instances of reasons underdetermination and the difficulty in determining a ‘best’ course of action is not due to lack of diligence, information, or computing power. but rather to the fact that multiple valid courses of action exist and no calculation can definitively reveal a ‘best’ one. And if, as I have tried to stress in 3.1, reasons underdetermination is a widespread phenomenon, then in order for the proximity scale of reasons to be a viable method for achieving Tracking it must find a way to integrate a solution to this problem.

To summarize, the three approaches discussed in this section provide valuable insights into the complexities of fulfilling the Tracking condition in MHC. Yet, as we have seen, none adequately addresses the problem of reasons underdetermination, leaving a critical gap. At the end of Sect. 3.1, we highlighted two methods for decision-making in the face of reasons underdetermination - Precisification and Commitments. Although there has been some preliminary discussion of their application in AI systems (Dobbe 2021; Goodman, 2021; Chang, 2024), these methods have not yet been explored in the context of MHC. Determining whether, and how, they could complement existing approaches to Tracking will need to be addressed in future research.

### Reasons underdetermination in tracing

In Sect. 2.2, we discussed the role of reasons in Tracing, arguing that Tracing seeks to mitigate or eliminate the emergence of responsibility gaps and that reasons play an important role in responsibility attribution. Our analysis examined three different notions of responsibility:

- **Responsibility as Accountability**: Who is to be blamed for the outcome?
- **Responsibility as Answerability**: Who can explain or justify why the action was taken?
- **Responsibility as Attributability**: Who made the decision?

In each of these three notions, reasons play a central role. For accountability, reasons are tied to the epistemic condition, which requires that an agent have sufficient knowledge or understanding of how the system functions in order to be held accountable, and thus blameworthy, for any wrongdoing. For accountability, reasons are tied to the epistemic condition, which requires that an agent have sufficient knowledge or understanding of how the system functions in order to be held accountable, and thus blameworthy, for any wrongdoing. Finally, in attributability, reasons connect to the agent’s practical identity: for an action to be attributable to an agent, the person must have made a value commitment, treating certain reasons as carrying special weight and guiding their actions accordingly.

After clarifying the role of reasons in each of these faces of moral responsibility, Sect. 2.2 discussed how the introduction of AI systems, especially DSSs, can potentially create responsibility gaps corresponding to each notion of responsibility. We concluded that appealing to some form of algorithmic transparency or explainability may help address these issues. However, this approach faces significant challenges. Designing a model to be inherently interpretable, often called a “Glass Box” model, is no small task, as creating interpretable models requires substantial computational resources and deep domain expertise (Rudin, 2019). Alternatively, techniques from Explainable AI (XAI) involve building a secondary model to interpret the results of the original model, but this raises concerns about whether the explanations truly reflect the original model’s decision-making process or are merely plausible narratives (Mittelstadt et al., 2019, Bell et al., 2022). Similar concerns arise with methods such as Chain-of-Thought (CoT), where an LLM is expected to describe the reasoning steps it followed to reach a given output. As Barez et al. recently noted, “CoTs can appear coherent and convincing, while not faithfully reflecting the true decision process of the model. This gap is not a rare anomaly” (Barez et al. 2025, 11).

Setting these technical difficulties aside, I want to highlight a more theoretical concern, namely that attempts to resolve responsibility gaps by appealing to access to reasons must also account for the problem of reasons underdetermination in its designed solution. Starting with the question of accountability, our concerns were that an agent acting on a recommendation of a DSS may lack the necessary knowledge for contesting or rejecting that recommendation. Even assuming we have the technical capability to explain a system’s outputs, the question remains: what should such an explanation include to enable accountability? Baum et al. (2022) argue that “a reason explanation needs to state explicitly what pieces of information served as reasons for or against a certain recommendation… [and] include the strengths of these reasons” (Baum et al., 2022, p. 23). They further emphasize that the explanation should be concise to avoid overwhelming the user or becoming difficult to understand.

However, this approach presents several challenges. First, if a system processes hundreds or thousands of variables to generate its output, there may be no single or small set of central reasons underlying the recommendation. Instead, each variable might contribute only marginally, making the emphasis on any specific reason potentially misleading or meaningless. Second, related to reasons underdetermination, the problem of incommensurability raises normative concerns about assigning precise weights to each reason. If a system is designed to do so, there is a risk that its evaluation will be overly artificial, imposing a normative structure on a decision that does not exist or producing reasoning that is not meaningful or interpretable to a human user. This recalls the comical discussion in the previous section about attempting to calculate the ‘perfect’ response to a case of mutiny.

Nevertheless, for the purposes of ensuring accountability, this may still be sufficient, since the most important aspect appears to be ensuring that a decision is not based on ‘bad’ or clearly wrong reasons. In contrast, answerability and attributability become much more complicated when reasons underdetermination is involved. Consider a simple, low-stakes example: an AI meal-planning system recommends serving a banana rather than a pear for dessert. When asked why it made this recommendation, the system responds:

‘The system chose the banana because of its creaminess’.

While this explanation may suffice to determine that choosing the banana is acceptable in this context, it does not meet the comparativist standards discussed in Sect. 3.1 and provides insufficient information for either answerability or attributability. Recall that the comparativist framework for rational choice holds that the rationality of a decision depends on an all-things-considered evaluation of the relevant comparative facts. To illustrate this, consider a more detailed version of the system’s non-comparativist explanation for choosing the banana over the pear:

**Table:** 

User:	Why did you select the banana and not the pear?
AI:	Because of the creaminess of the banana.
User:	You mean the creaminess of the banana is better than the sweetness of the pear?
AI:	No, the creaminess of the banana makes it good so I selected it.
User:	Ahh I see, you determined that soft fruits are better than crunchy fruits?
AI:	No! What don’t you understand? The creaminess of the banana is a good making feature of this fruit and gives me a reason to select it, and so I selected it.
User:	But doesn't the sweetness of the pear give you a reason to select it also? Why didn’t you select the pear?
AI:	Yes, you are of course right, there are good reasons to select the pear, but I selected the banana, not because it is better, or because I think you would like it more, or even because the pear is not creamy, but simply because the banana is creamy.
User:	I don’t understand.

The user’s perplexity in this example is entirely understandable. While selecting the banana for its creaminess may explain the reason behind that choice, it does not justify or defend its rationality. Reasons underdetermination complicates matters further, as it often requires choosing among multiple possible courses of action, each potentially very different, without a rational basis for asserting that one option is better than the others. While this may seem trivial when deciding between a banana and a pear, it becomes critically important in high-stakes scenarios, such as approving a business loan, selecting a candidate for a position, or determining which patient should receive an organ transplant first (Sinnott-Armstrong & Skorburg, 2021).

In terms of answerability, consider an AI system recommending that an organ be given to patient A rather than patient B. If this situation involves reasons underdetermination, it means the system could have reasonably justified selecting either patient but ultimately chose A. For the doctor to be answerable for this recommendation, she would require a specific explanation: why did the system select A? If the system merely cites a single factor—such as the patient’s age, number of children, or overall health—this would not suffice. Such a response overlooks the fact that multiple factors also favor patient B and that the balance of reasons does not determine a clear choice. Some theorists argue that AI systems should be designed to avoid making recommendations in situations of reasons underdetermination altogether (Goodman, 2021). While this would prevent the system from issuing decisions without a definitive basis, it could severely limit the system’s usability if such situations are common. Alternatively, if designers implement mechanisms to resolve these cases, the doctor would need clear, detailed information on the trade-offs embedded in the system to understand and justify the recommendation effectively.

The same issue also arises with regard to attributability, which, according to Zeiser, concerns whether the “relevant value-judgements expressed in a decision… reflect value-judgements of the human decision-maker who made the decision” (Zeiser, 2024, p. 14). Simply endorsing a recommendation from a DSS can create a clear attributability gap if the decision-maker cannot access the value judgments that led to that specific output. This raises two key concerns. First, as with answerability, the system’s explanation should include all relevant value judgments, both those generated by the system and those embedded in it by design. Second, in cases of reasons underdetermination, the decision-maker may experience perplexity, as the reasons allow for multiple reasonable options. This may discourage them from contesting the system’s recommendation, resulting in a loss of agency and diminished ownership over their decisions (Danaher, 2022).

In sum, this section has aimed to show that while providing a reasons-based explanation for why a system acted as it did may initially appear to be a straightforward solution for achieving Tracing and avoiding responsibility gaps, there are numerous challenges that such an approach must address, many of which are amplified by the widespread existence of reasons underdetermination. As such, even though reasons play a central role in responsibility attribution, simply providing access to a system’s reasoning is far from sufficient for achieving Tracing. Addressing these challenges and developing effective methods for operationalizing Tracing in light of reasons underdetermination will require further careful investigation in future research.

## Conclusion

The MHC framework has the potential to play a pivotal role in the ongoing effort to design AI systems that are safe and responsible. By establishing standards for evaluating systems, it serves both as a guide for developers and as a benchmark for assessing AI design. In this paper, I have highlighted a significant theoretical issue that remains unaddressed within the MHC framework: the pervasive phenomenon of reasons underdetermination. I have discussed its implications for both the Tracking and Tracing conditions and argued that this underexplored challenge must be addressed in future developments of the framework.

Moving forward, research will need to focus on two central aspects. First, it is necessary to establish the widespread existence of reasons underdetermination and its concrete implications for AI system design and use, ideally through real-world examples and case studies. Second, potential solutions to this problem must be explored. This paper has outlined three possible avenues: designing algorithmic systems that detect cases of reasons underdetermination and flag them for human intervention; adopting existing theoretical methods, such as precisification or commitments, and developing system designs that incorporate these approaches; and, finally, considering alternative strategies to operationalize MHC that do not center reasons as the primary variable. As noted above, this final approach may offer a viable path for operationalizing the Tracing condition in certain AI systems that do not require a close understanding of the reasoning behind their outputs. However, for Tracking and for operationalizing Tracing across many systems, particularly DSSs, engaging with reasons appears unavoidable. Consequently, for the MHC framework to continue gaining traction, it is essential to develop strategies to overcome the challenge posed by reasons underdetermination.

## Data Availability

No datasets were generated or analysed during the current study.

## Footnotes

1 I would like to thank an anonymous reviewer whose feedback on this section enabled me to revise it in a more nuanced and, I believe, more accurate way.

2 There is a large literature which debates the relationship between reasons, values, and ought in relation to practical reason and action. I do not go into this debate here as it would unnecessarily complicate the matter being discussed. For further discussion on these issues see Raz, 1999.

3 Another layer of complexity arises from the possibility that an action may be, normatively speaking, the right thing to do, yet the agent is motivated by unrelated, or even objectionable, reasons. A classic example is donating money to a worthy cause solely for the sake of social recognition. Likewise, imagine a case in which Herbert’s DSS recommends not hiring April on the basis of her skin color, but it later emerges that there were independent and legitimate reasons not to hire her, that she had forged her diploma. I set such cases aside as the former raises the complex issue of the moral worth of reasons (for a detailed discussion, see Markovits, 2010), while the latter represents instances where the right action is reached only coincidentally. I thank an anonymous referee for raising this point.
